# Increased Resting-State Functional Connectivity in the Cingulo-Opercular Cognitive-Control Network after Intervention in Children with Reading Difficulties

**DOI:** 10.1371/journal.pone.0133762

**Published:** 2015-07-21

**Authors:** Tzipi Horowitz-Kraus, Claudio Toro-Serey, Mark DiFrancesco

**Affiliations:** 1 Pediatric Neuroimaging Research Consortium, Cincinnati Children’s Hospital Medical Center, Cincinnati, Ohio, United States of America; 2 Reading and Literacy Discovery Center, Cincinnati Children’s Hospital Medical Center, Cincinnati, Ohio, United States of America; University of British Columbia, CANADA

## Abstract

Dyslexia, or reading difficulty, is characterized by slow, inaccurate reading accompanied by executive dysfunction. Reading training using the Reading Acceleration Program improves reading and executive functions in both children with dyslexia and typical readers. This improvement is associated with increased activation in and functional connectivity between the anterior cingulate cortex, part of the cingulo-opercular cognitive-control network, and the fusiform gyrus during a reading task after training. The objective of the current study was to determine whether the training also has an effect on functional connectivity of the cingulo-opercular and fronto-parietal cognitive-control networks during rest in children with dyslexia and typical readers. Fifteen children with reading difficulty and 17 typical readers (8-12 years old) were included in the study. Reading and executive functions behavioral measures and resting-state functional magnetic resonance imaging data were collected before and after reading training. Imaging data were analyzed using a graphical network-modeling tool. Both reading groups had increased reading and executive-functions scores after training, with greater gains among the dyslexia group. Training may have less effect on cognitive control in typical readers and a more direct effect on the visual area, as previously reported. Statistical analysis revealed that compared to typical readers, children with reading difficulty had significantly greater functional connectivity in the cingulo-opercular network after training, which may demonstrate the importance of cognitive control during reading in this population. These results support previous findings of increased error-monitoring activation after reading training in children with dyslexia and confirm greater gains with training in this group.

## Introduction

Reading difficulty (RD), or dyslexia, is a deficit in acquiring fluent reading skills despite remedial intervention and repeated exposure to written language [1]. Individuals with RD also share deficits in other cognitive domains, such as in executive functions (EF) [2–8]. More specifically, it has been reported that individuals with RD share difficulties in attention [9–11] speed of processing [12], inhibition [3], working memory [5, 13], and set-shifting [14],as well as in error monitoring in the linguistic [15, 16] and non-linguistic domains [14, 17]. The findings that individuals with RD share deficits in cognitive abilities underlying reading (i.e., in EF) have been supported by several neuroimaging studies. Vogel and colleagues suggest that the part of the ventral stream related to orthographic processing (i.e., the ‘visual word form area’ or VWFA) is functionally connected to the dorsal attention network, as determined during resting-state functional magnetic resonance imaging (fMRI) [18, 19]. Other neuroimaging studies also support the role of visual attention in particular [20] and EF in general [21] in individuals with RD.

The ‘dual-networks top-down’ model [22] is a functional connectivity-based model that proposes two cognitive-control/EF networks with different neuroanatomical correlates; 1) the fronto-parietal network, which is a rapid adaptive-control network that allocates attention to a cue and 2) the cingulo-opercular network, which is a set-maintenance network that maintains task goals, sustains adjustments for feedback control, and monitors errors (also see [23] for the functional organization of these regions). The connectivity within these two networks increases throughout development [24] and both networks are engaged during reading [25]. Due to reports related specifically to altered EF that form the basis of reading (i.e. slower speed of processing [12] and impaired visual attention [20] and error monitoring [15]) in individuals with dyslexia, there is a particular interest in looking at the differences in functional connectivity in cognitive-control networks related to these abilities in this population. Another intriguing question is whether a reading intervention program targeting these abilities specifically will affect functional connectivity of the fronto-parietal and cingulo-opercular networks.

Reading is a higher-order cognitive ability that relies on phonology, orthography, and semantics, as well as EF (i.e., working memory [26]], speed of processing [12], and switching/shifting attention and inhibition [27]). A recent causality model confirmed the close relationship between EF and academic achievement, which is highly dependent on reading, and showed a high correlation between these two measures in 2.5–13 year-old children with absence epilepsy [28].

The Reading Acceleration Program (RAP) is a computer-based program designed to improve reading fluency through basic EF-principles [29]. The RAP manipulates the rate of the reading materials presented to each individual based on the participant’s own reading rate in a time-constrained manner. It has been suggested that the RAP minimizes the discrepancy between potential reading abilities and actual reading performance in individuals with either intact or impaired reading [1]. Reading speed, word-decoding accuracy, and reading comprehension improve after the RAP training in both children with RD and typical readers (TRs) in different age groups (young readers [30–36]] and adult readers [1, 37] and in different orthographies (Hebrew [33, 34, 37], English [36, 38, 39], German [40], and Dutch [41].

Due to the specific manipulation of the RAP in forcing the reader to process more letters in a given time and track the deleted letters with their eyes, one possible mechanism for the effect of the RAP is through a direct effect on the executive system in individuals with RD [35, 36]. This is consistent with previous EEG results showing greater error-detection activation related to the anterior cingulate cortex (part of the cingulo-opercular network) for reading errors in both children and adults with RD [33, 42]. A recent fMRI study demonstrated that following 4 weeks of the RAP training, a greater activation of the fusiform gyrus occurred, as well as increased involvement of regions related to EF (i.e., dorsolateral prefrontal cortex, inferior frontal gyrus, medial frontal gyrus, and anterior cingulate cortex) [36]. In support of pioneering work demonstrating a positive relationship between task activation and functional connectivity during rest [43], an increased functional connectivity between the fusiform gyrus and the anterior cingulate cortex has been observed in individuals with RD after the RAP training, both during a reading task [35] and during rest using an independent component analysis (ICA) approach [44]. Results of that study also indicated an increased functional connectivity of components related to EF (composed of the dorso-lateral prefrontal cortex) in children with RD during rest following the RAP training [44]. We suggest that the RAP training improves the functional connections between EF-related components and the visual-processing components even during resting state condition, which may indicate the involvement of the cognitive-control networks in reading improvement. These results are consistent with previous findings indicating an increased functional connectivity in individuals with RD between the left VWFA (BA 37) related to orthographic processing and both frontal regions related to EF (medial frontal gyrus; BA 10) and other reading regions (middle occipital regions; BA 18,19) following reading intervention, as compared with TRs [45]. Despite these consistent findings, the specific effect of the RAP training on the dual-mode networks during a resting condition in individuals with RD and TRs is yet to be determined.

The goal of the current study was to specifically test the effect of the RAP training on functional connectivity of the cognitive-control networks (described in [22]). To do this, 15 children with RD and 17 TRs were trained with the RAP and scanned using a resting-state fMRI paradigm both before and after training. We hypothesized that in support of our previous findings of a greater gain from training with the RAP in individuals with RD, children with RD would demonstrate a greater increase in functional connectivity, specifically in global efficiency, in both the cingulo-opercular and fronto-parietal networks. Global efficiency is a measure of functional integration [46] that represents the average inverse shortest path length between all nodal pairs in the network. It is an example of a graph metric, like the clustering coefficient and characteristic path length, which are useful for characterizing the global organization of large-scale networks ([46]; for other studies using these measures see also [47, 48]). Our expectation of elevated functional integration was based on our previous findings showing an increased blood-oxygen-level-dependent (BOLD) signal in the anterior cingulate cortex following the RAP training [36] as well as the critical role of this brain region in reading [38]. We also based our assumption on previous studies showing a change in functional connectivity during rest in children with dyslexia that was accompanied by reading improvement [45]. We also hypothesized that the combined increase in global efficiency in the fronto-parietal and cingulo-opercular cognitive-control networks would be positively correlated with greater reading improvement in children with RD.

## Methods and Materials

### Participants

Fifteen children with RD (mean age = 10.27 years, SD = 1.48 years; 8 female) and 17 TRs (mean age = 9.77 years, SD = 1.44 years; 8 female) participated in the current study, all matched for age (t(30) = -0.365, *P* = 0.718). All participants were within the normal range of nonverbal IQ (mean = 102.71, SD = 6.45), with no significant differences between the groups [children with RD: 102.27±5.71, TRs: 103.13±7.247; t(30) = 0.397, *P* = 0.33] as measured by the Test of Nonverbal Intelligence, Third Edition (TONI-3; [49]). No differences were found in general verbal ability (vocabulary subtest from the Wechsler Intelligence Scores for Children: [50]) between the children with RD and TRs [children with RD: 10.87±2.82, TRs: 9.66±3.12; t(30) = 1.152, *P* = 0.258]. All 32 participants underwent baseline behavioral and neuroimaging assessment (Test 1) followed by 4 weeks of the RAP training, with follow-up behavioral and neuroimaging assessment (Test 2).

All participants were Caucasian, native English speakers with average socioeconomic status, as reported by the families. Participants were right-handed, displayed normal or corrected-to-normal vision in both eyes, and had normal hearing. None had a history of neurological or emotional disorders, and no differences were found between the two reading groups in attention ability [measured by the Connors questionnaires ([51]; self-report: t(30) = 0.677, *P* = 0.49; and parents’ report: t(30) = 0.246, *P* = 0.236]. Participants were recruited from posted ads and through commercial advertisements. All participants gave informed written assent and their parents provided informed written consent prior to inclusion in the study, and all were compensated for their participation. The Cincinnati Children’s Hospital Medical Center (CCHMC) Institutional Review Board approved the study.

### Diagnostic assessment

Children with RD had either received previous diagnoses or parents had reported their children as having difficulty with reading. In the first meeting (Test 1), we confirmed the existence of RD by using a battery of normative reading tests in English. Inclusion criteria for the RD group were a score of 25 percent or below for a phonemic awareness task involving word reading and decoding abilities. The reading battery included a) words reading accuracy/orthography: the Test of Words Reading Efficiency (TOWRE; [52]), b) decoding: the Pseudowords Reading Efficiency subtest from the TOWRE [52], and c) phonemic awareness: the Elision subtest from the Comprehensive Test of Phonological Processing (CTOPP; [53]). Participants in the TRs group were age-matched students who volunteered for the study and had fluent and accurate reading (according to norms). Reading by TRs was examined using the same tests used to evaluate the children with RD. The results from these diagnostic reading tests also were used as baseline reading measures before intervention (Test 1). The study was carried out in the Pediatric Imaging Research Consortium (PNRC) at CCHMC in Cincinnati, Ohio.

### Measures

#### Reading measures

To measure the effect of the RAP training on phonemic awareness, automaticity in reading, and contextual reading fluency and comprehension, we used the Elision subtest (from the CTOPP), the TOWRE word and pseudoword reading tests, and the reading fluency (i.e., speed) and reading comprehension measures exported from the RAP before training (Test 1) and following 4 weeks of training (Test 2).

#### Executive functions measures

Executive functions were measured using several sub-tests from different batteries: a) attention using speed and accuracy subtests from the TEA-Ch battery ([54]; Sky Search subtest), b) fluency abilities using the Delis-Kaplan Executive Functions System (D-KEFS; [55]), c) speed of processing using the “object naming” subtest from the CTOPP [53], and d) switching abilities using the Wisconsin Card Sorting Task [56] and e) inhibition abilities using the “Stroop” subtests from the D-KEFS [55]. The behavioral data (reading and EF) acquisition lasted approximately 2 hours.

#### Behavioral data analysis

Behavioral data were analyzed using SPSS 11 (SPSS Statistics, version 11, IBM, Armonk, New York, USA). Two-sample *t*-tests were used to examine group differences in age and IQ measures. To verify the effect of the RAP on reading and EF measures in the two groups, several 2 × 2 Repeated-Measures Analyses of Variance (RM-ANOVA) for Group (children with RD, TRs) and RAP training (Test 1 –before intervention, Test 2 –follow-up 4 weeks after training) were used for each of the described measures. To determine the relationship between the improvements in reading and EF following training, we also performed an entire-sample Pearson correlation between the gain in reading (i.e., the difference between reading scores in Test 1 and Test 2) and the EF measures.

### Functional MRI

#### Procedure

We assessed the effect of the RAP training on resting-state global efficiency in all participants using a resting-state fMRI protocol. Participants were asked to look at a grey cross in the center of a projector screen for 5.5 minutes (i.e., a resting-state condition) and to avoid sleeping or closing their eyes. Participants performed this scan before and following the RAP training (approximately 5 weeks apart).

#### MRI acquisition and data preprocessing

All images were acquired on a Philips Achieva 3T MRI scanner (Philips Medical Systems, Best, The Netherlands). A T2*-weighted, gradient-echo, echo planar imaging (EPI) sequence was used with fMRI parameters: TR/TE = 2000/38 msec, matrix size = 64 × 64, slice thickness = 5 mm, resulting in a voxel size = 4 × 4 × 5 mm^3^. During the resting-state scan, 165 whole-brain volumes were acquired for a total imaging time of 5.5 minutes. The initial 10 time points acquired were discarded to allow for T1 relaxation equilibrium. In addition, a high-resolution T1-weighted 3D anatomical scan was acquired using an inversion recovery (IR)-prepared turbo gradient-echo acquisition protocol with a spatial resolution of 1 × 1 × 1 mm^3^.

Participants were acclimated and desensitized to the scanner to condition them for comfort during imaging (see [57] for details). Head motions were controlled using elastic straps that were attached to either side of the head-coil apparatus.

#### MRI data analysis

During image reconstruction, a multi-echo reference scan was initially used to correct for Nyquist ghosts and geometric distortion due to B0 field inhomogeneity. Reconstructed fMRI data were then spatially pre-processed using SPM8 software (www.fil.ion.ucl.ac.uk/spm/), including slice-timing correction, realignment for motion correction, coregistration of the anatomical image to the mean aligned functional image, segmentation by gray matter, white matter, and cerebrospinal fluid tissue classes, normalization of all images to the Montreal Neurological Institute (MNI) space, and spatial smoothing with an 8-mm full width at half-maximum (FWHM) Gaussian kernel. Motion was corrected using pyramid co-registration [58] in SPM8. We performed 3-dimentional affine transformation to align the volumes. This resulted in six motion parameters; three translational and three rotational. No significant differences in motion parameters were found for each of the six motion parameters (see Table 1). In addition, time points with excessive motion were rejected from the post processing pipeline. We used a mutual information cost function for rejecting motion-corrupted frames of fMRI data as previously described [59]. All data met the criterion of median voxel displacement < 2 mm in the center of the brain.

**Table 1:** Comparison of the baseline mean motion of individuals with reading difficulty and typical readers in translational and rotational orientations.

Orientation	Axis	Group	Mean (SD)	t(30)
**Translational**	X	RD	-0.075 (0.161)	1.260 (*P* = 0.217)
		TRs	-0.153 (0.189)	
	Y	RD	-0.208 (0.216)	.076(*P* = 0.94)
		TRs	-0.217 (0.42)	
	Z	RD	0.258 (0.585)	-1.4(*P* = 0.172)
		TRs	0.771 (1.342)	
**Rotational**	X	RD	-0.002 (0.011)	.128(*P* = 0.899)
		TRs	-0.002 (0.017)	
	Y	RD	-0.002 (0.004)	.056(*P* = 0.956)
		TRs	-0.002 (0.011)	
	Z	RD	-0.001 (0.001)	1.011(*P* = 0.328)
		TRs	-0.005 (0.022)	

RD, children with RD (reading difficulty—dyslexia); TRs, typical readers; t, independent *t*-test, *P* = significance. Results are presented as mean (standard deviation).

Following spatial pre-processing, the resting-state data were fed into CONN [60], a functional connectivity toolbox for Matlab (The Mathworks, Natick, MA). Additional preprocessing under the anatomical component-based noise-correction framework (aCompCor) [61] included extraction of the first five principle eigenvariates of the BOLD time-courses from white matter and cerebral spinal fluid (CSF) regions for use as regressors in the analysis to remove signal variation associated with these non-cortical regions. In addition, the six motion parameters for each session, together with their first derivatives, were regressed out of the voxel time series. Finally, the voxel time series data were band-pass filtered between 0.008 and 0.2 Hz, as recommended by Baria and colleagues [62]. Functional connectivity between pairs of target regions of interest (ROI) was calculated as the correlation coefficient for the average voxel signal per ROI pair.

#### Target regions of interest

To determine the functional connectivity within the cingulo-opercular and fronto-parietal networks, we first identified ROI defining the nodes of these networks based on coordinates reported by Dosenbach and colleagues [22]. ROI coordinates are listed in Table 2. ROI masks were generated using the WFU pick atlas toolbox [63] for SPM8 (http://fmri.wfubmc.edu/research/PickAtlas). Each region was a spherical seed (10 mm radius in 2 mm standard space). MNI coordinates were modeled (after [22] and have been described previously ([64, 65].

**Table 2:** Regions of interest groups with corresponding coordinates.

Cingulo-Opercular Network	*X*	*Y*	*Z*
Left anterior Prefrontal Cortex [aPFC (L)]	-28	51	15
Right anterior Prefrontal Cortex [aPFC (R)]	27	50	23
Left Lateral anterior Insula / frontal Operculum [Lateral aI fO (L)]	-51	18	13
Right Lateral anterior Insula / frontal Operculum [Lateral aI fO (R)]	45	23	-4
Left Medial anterior Insula / frontal Operculum [Medial aI fO (L)]	-33	24	1
Right Medial anterior Insula / frontal Operculum [Medial aI fO (R)]	33	25	-1
Left anterior Insula / frontal Operculum [aI fO (L)]	-35	14	5
Right anterior Insula / frontal Operculum [aI fO (L)]	36	16	4
Dorsal anterior cingulate / medial superior Frontal Cortex [dACC msFC]	-1	10	46
Fronto-Parietal Network	*X*	*Y*	*Z*
Left dorso-lateral Prefrontal Cortex [dlPFC (L)]	-43	22	34
Right dorso-lateral Prefrontal Cortex [dlPFC (R)]	43	22	34
Left inferior Parietal Lobule [IPL (L)]	-51	-51	36
Right inferior Parietal Lobule [IPL (R)]	51	47	42
Left Intraparietal Sulcus [IPS (L)]	-31	-59	42
Right Intraparietal Sulcus [IPS (R)]	30	-61	39
Left Precuneus [Precuneus (L)]	-9	-72	37
Right Precuneus [Precuneus (R)]	10	-69	39
Mid Cingulate Cortex [mCC]	0	-29	30

#### Functional connectivity analysis

Functional network connectivity analysis, including calculation of global efficiency, was performed for each network separately (i.e., cingulo-opercular, fronto-parietal) for each testing time (Test 1, Test 2). The global efficiency was calculated in CONN using the formula (from [66]):

$$E=\;\frac{1}{n}\sum_{i\in N}\;E_{i}\;=\;\frac{1}{n}\sum_{i\in N}\;\frac{\sum_{j\in N,j\neq i}d_{ij}^{-1}}{n-1}$$

where *E*_*i*_ is the efficiency of node *i*, *n* is the number of network nodes, *N* is the set of all network nodes, and *d*_*ij*_^*-1*^ is the inverse shortest pathlength between nodes *i* and *j*.

To measure the effect of intervention comparing the two groups and the two networks, a 2 × 2 × 2 RM-ANOVA for Group (children with RD, TRs), Network (cingulo-opercular, fronto-parietal), and testing time (Test 1, Test 2) was performed. To measure the gain from intervention in each group, we performed the following paired and independent *t*-tests comparing global efficiency values: children with RD in Test 2 > Test 1; TRs in Test 2 > Test 1. Results were corrected for false discovery rate (FDR) and significance was set at *P*<0.05.

#### Correlation of global efficiency with behavioral scores

To determine the associations between the change in global efficiency and the change in reading outcomes, a Pearson correlation between these measures was performed within each group.

Since we were interested in defining the relationship between the change in functional connectivity in the cognitive-control networks and the change in reading measures and EF, we also correlated the “gain” measure with the gain of these behavioral measures using a Pearson correlation (an overall of 13 correlations per network and 13 correlations for the combined gain in global efficiency for both the cingulo-opercular and fronto-parietal networks). Data was corrected for multiple comparisons using a Bonferroni correction.

### Reading Acceleration Program

#### Stimuli

The RAP bank of 1500 sentences was composed of moderate- to high-frequency words in the English language (http://www.wordfrequency.info/). Each stimulus was a sentence with a multiple-choice question followed by four possible answers. Each sentence length was 9–12 words, composed of 45–70 letters with a letter width of 5 mm, and extending over one to two lines with 18 mm between lines. Each sentence was presented once during the entire training. The sentences have been tested and verified for their level of difficulty in previous studies [1].

#### Training procedure

Reading training was administered via the internet using a computer in the participant’s home. The primary investigator monitored training by remote access to the training records. The participants were trained for four weeks, five times each week and 15–20 minutes per session, for a total of 20 sessions reading a different set of 50 randomly presented sentences in each session. The initial and final reading pace and comprehension were measured by the evaluation mode of the RAP [37] which measures these variables in a self-paced reading condition (see *Behavioral Data Analysis* section).

The duration of a sentence on the screen was calculated individually for each participant based on the diagnostic mode of the RAP (see *Presentation rate* section). The duration was controlled by text erasure starting from the beginning of the sentence and advancing at a given per-character rate. All participants were presented with the same sets of sentences in the same order. They were instructed to read the sentence silently and while doing so, the sentence disappeared from the computer screen and a multiple-choice comprehension question appeared and remained on the screen until the participant responded. They were instructed to choose the correct answer by pushing the corresponding number on the numeric key pad of the computer. The disappearance of the question from the computer screen prompted presentation of the next sentence.

#### Presentation rate

The initial text erasure rate was determined specifically for each participant, based on a pre-test evaluation mode administered prior to training. This evaluation mode consisted of 12 sentences and 12 multiple-choice questions [67]. The mean reading rate [milliseconds (ms) per letter] for the sentence correctly answered determined the initial erasure rate of the RAP for that participant.

#### Accelerated training condition

In the first training session, 50 sentences were presented consecutively on the screen. The letters in each sentence disappeared one after the other, according to the mean reading time (ms per letter) recorded on the pre-test. Following the disappearance of the sentence from the computer screen, participants were instructed to answer the question at a self-paced rate. The per-letter “erasure rate” decreased from one sentence to the next in steps of 2 percent [30, 31] and in a staircase-like procedure, during which the “erasure rate” increased only when the participants’ answers to the probe questions were correct on 10 consecutive sentences.

## Results

### Effect of the RAP training on behavioral measures

#### Reading measures

At baseline, children with RD had decreased phonemic awareness, word, pseudoword, and contextual fluency, and reading comprehension scores compared to those for TRs. An overall effect of intervention (i.e., main effect of Test) was observed in all of these measures, except phonemic awareness. Children with RD had globally improved word and pseudoword reading as well as better reading fluency and comprehension abilities after training (Table 3).

**Table 3:** Reading behavioral measures before and after reading intervention in children with reading difficulty and typical readers.

Measure		RD		TRs		F	*t*-test *P* value	Contrast
		Test 1 (A)	Test 2 (B)	Test 1 (C)	Test 2 (D)			
Reading	Automaticity in word reading (TOWRE; percentiles)	13 (18.733)	21 (22.7)	53 (21.69)	73.94 (16.67)	Test: F(1,30) = 19.638, *P*<0.001, η^2^ = 0.404; Group: F(1,30) = 51.890, *P*<0.001, η^2^ = 0.642; Test × Group: F(1,30) = 3.907, *P* = 0.058, η^2^ = 0.119	-1.47*	B > A
							-5.42***	D > C
							-5.52***	C > A
	Automaticity in pseudo word reading (TOWRE; percentiles)	12.71 (17.03)	21.71 (21.699)	56.88 (19.17)	72.29 (19.76)	Test: F(1.30) = 13.207, *P*<0.001, η^2^ = 0.313; Group: F(1,30) = 58.760, *P*<0.001, η^2^ = 0.670; Test × Group: F(1,30) = 0.911, *P* = 0.348, η^2^ = 0.030	-1.45*	B > A
							-4.65***	D > C
							-7.07***	C > A
	Global reading efficiency (TOWRE, SWE+PWE, scaled score)	75 (13.13)	80.27 (10.7)	102.61 (8.97)	111.53 (10.53)	Test: F(1,30) = 34.740, *P*<0.001, η^2^ = 0.53; Group: F(1,30) = 62.83, *P*<0.001, η^2^ = 0.67; Test × Group: F(1,30) = 3.396, *P* = 0.06, η^2^ = 0.102	-2.41*	B > A
							-6.89***	D > C
							-6.86***	A < C
	Reading fluency (from RAP, msec/letter)	172.06 (67.11)	127.96 (45)	105.62 (41.28)	72.35 (20.09)	Test: F(1,30) = 11.627, *P*<0.01, η^2^ = 0.293; Group: F(1,30) = 24.833, *P*<0.001, η^2^ = 0.470; Test × Group: F(1,30) = 0.227, *P* = 0.637, η^2^ = 0.008	1.95*	A < B
							4.25**	C < D
							3.21**	A < C
	Reading comprehension (from the RAP, accuracy percentages)	62.49 (4.43)	88.15 (6.1) 96.15 (5.61)	96.57 (6.1)	97.0 (5.1)	Test: F(1,30) = 110.27, *P*<0.001, η^2^ = 0.797; Group: F(1,30) = 154.62, *P*<0.001, η^2^ = 0.847; Test × Group: F(1,30) = 103.45, *P*<0.001, η^2^ = 0.787	-13.64***	A > B
							-0.25, ns	C > D
							-18.32***	A < C
	Phonemic awareness (CTOPP, scaled scores)	7.47 (3.46)	8 (3.16)	11.76 (1.6)	12 (2.15)	Test: F(1,30) = 0.799, *P* = 0.37, η^2^ = 0.026; Group: F(1,30) = 24.613, *P*<0.001, η^2^ = 0.451; Test × Group: F(1,30) = 0.12, *P* = 0.731, η^2^ = 0.04	-0.66,ns	B > A
							-0.61,ns	D > C
							-4.00***	C > A

RD, children with RD (reading difficulty—dyslexia); TRs, typical readers; F, variance for the RM-ANOVA; TOWRE, Test of Word Reading Efficiency; RAP, Reading Acceleration Program; CTOPP, Comprehensive Test of Phonological Processing. Results are presented as mean (standard deviation).

*, *P*<0.05;

**, *P*<0.01;

***, *P*<0.001.

#### Executive function measures

Children with RD had lower EF scores than TRs in most of the variables examined: attention (time), fluency, inhibition, switching, and speed of processing. An overall effect of Test was found for most EF; accuracy and speed in the visual attention task, speed of processing, and inhibition. Specifically, children with RD had improvement after intervention in attention (time and accuracy), inhibition, speed of processing, switching, and overall EF as measured by the Wisconsin task (Table 4).

**Table 4:** Executive functions behavioral measures before and after reading intervention in children with reading difficulty and typical readers.

Measure		RD		TRs		F	*t*-test *P* value	Contrast
		Test 1 (A)	Test 2 (B)	Test 1 (C)	Test 2 (D)			
Executive functions	Attention, time (TEA-Ch Sky Search time per target, scaled score)	7.36 (3.6)	9.64 (2.56)	8.71 (2.08)	10.41 (2.59)	Test: F(1,30) = 8.281, *P*<0.01, η^2^ = 0.222; Group: F(1,30) = 2.291, *P* = 0.141, η^2^ = 01; Test × Group: F(1,30) = 0.175, *P* = 0.679, η^2^ = 0.006	-3.98**	B > A
							-2.02*	D > C
							-0.83 ns	C > A
	Attention, accuracy (TEA-Ch Sky Search attention score, scaled score)	7.15 (2.44)	10.62 (1.89)	8.20 (2.27)	9.27 (2.21)	Test F(1,30) = 12.622, *P*<0.01, η^2^ = 0.327; Group F(1,30) = 0.076, *P* = 0.785, η^2^ = 0.003; Test × Group: F(1,30) = 0.3530, *P* = .0072, η^2^ = 0.120	-3.90**	B > A
							-1.18 ns	D > C
							-1.30 ns	C > A
	Speed of processing (from the naming object subtest, CTOPP, in seconds)	86.1 3(16.64)	44.53 (12.2)	73 (24.61)	71 (24.36)	Test: F(1,30) = 227.7 *P*<0.001, η^2^ = 0.884; Group: F(1,30) = 4.903, *P*<0.05, η^2^ = 14; Test × Group: F(1,30) = 0.148, *P* = 0.703, η^2^ = 0.005	2.23*	A > B
							0.889 ns	C>D
							1.742, *P* = 0.08	A>C
	Overall EF (Wisconsin Non-Perseverative errors, percentile)	64.07 (20.82)	80.33 (17.14)	75.07 (19.19)	73.64 (26.44)	Test: F(1,27) = 2.040, *P* = 0.165, η^2^ = 0.070; Group: F(1,27) = 0.135, *P* = 0.717, η^2^ = 0.005; Test × Group: F(1,27) = 2.902, *P* = 0.100, η^2^ = 0.097	-2.48*	B > A
							-.18 ns	C > D
							-1.48 ns	C > A
	Fluency (F,A,S letters, D-KEFS, number correct, scaled score)	8.43 (2.87)	9.93 (2.55)	11.27 (2.52)	11.33 (3.08)	Test: F(1,30) = 1.369, *P* = 0.252, η^2^ = 0.048; Group: F(1,30) = 7.351, *P*<0.05, η^2^ = 0.214; Test × Group: F(1,30) = 1.146, *P* = .0294, η^2^ = 0.041	-1.62 ns	B > A
							-0.07 ns	D > C
							-2.99**	C > A
	Inhibition (D-KEFS Stroop, scaled score)	6.71 (2.43)	10.29 (3.12)	10.00 (3.04)	10.47 (3.35)	Test: F(1,30) = 4.822, *P*<0.05, η^2^ = 0.152; Group: F(1,30) = 7.303, *P*<0.05, η^2^ = 0.213; Test × Group: F(1,30) = 2.851, *P* = 0.103, η^2^ = 0.095	-2.87*	B > A
							-0.35 ns	D > C
							-3.26**	C > A
	Switching (D-KEFS Stroop, scaled score)	7.07 (2.49)	10.57 (2.13)	11.40 (2.38)	10.67 (3.86)	Test; F(1,30) = 2.921, *P* = 0.099, η^2^ = 0.098; Group; F(1,30) = 11.054, *P*<0.01, η^2^ = 0.290; Test × Group; F(1,30) = 6.838, *P*<0.05, η^2^ = 0.202	-4.06*	B > A
							0.55 ns	C > D
							-5.30***	C > A

RD, children with RD (reading difficulty—dyslexia); TRs, typical readers; F, variance for the RM-ANOVA; TEA-Ch, Test of Everyday Attention for Children; EF, executive functions; D-KEFS, Delis-Kaufman Executive Functions System. Results are presented as mean (standard deviation).

*, *P*<0.05;

**, *P*<0.01;

***, *P*<0.001.

#### Correlation between reading and executive function measures

A Pearson correlation for the entire sample between gain in 1) single word reading, 2) pseudowords reading, 3) an overall reading efficiency, 4) contextual reading comprehension and 5) contextual reading with all EF in the entire sample was performed (seven correlations per these five conditions). Following correction for multiple comparisons per condition, the analysis revealed:

1. a positive correlation between the gain in word reading (the difference in TOWRE score for word reading in Test 1 and Test 2) and the gain in each of the EF abilities; object-naming time (r = 0.344, *P* = 0.08) and standard scores for fluency (r = 0.396, *P*<0.05), inhibition (r = 0.45, *P*<0.05), and switching (r = 0.348, *P* = 0.08). Therefore, greater improvement in word reading ability was associated with better speed of processing, fluency, inhibition, and switching abilities.
2. no significant correlation between pseudword reading (the difference in PWE from the TOWRE score for pseudoword reading in Test 1 and Test 2) and the gain in each of the EF abilities.
3. a positive correlation between overall reading ability (i.e., the difference in overall word and pseudoword reading scaled scores from the TOWRE) and the gain in visual attention accuracy scores (scales score from the Sky Search subtest in the TEA-Ch battery) (r = 0.327, *P* = 0.08). Therefore, greater gain in word and pseudoword reading ability was associated with greater gain in visual attention in both children with RD and TRs.
4. an improved reading comprehension (as measured by the RAP) and better attention ability (as measured by the Sky Search subtest; attention accuracy; r = 0.422, *P*<0.05) and switching abilities (as measured by the Stroop subtests: r = 0.37, *P*<0.05). Therefore, higher reading-comprehension scores were associated with better attention and switching abilities.
5. no significant correlations between the gain in contextual reading fluency or comprehension and gain in EF.

### MRI data analysis

#### The effect of the RAP training on a resting-state functional connectivity in the cognitive-control networks

Functional connectivity was characterized by the global efficiency measure exported in the analysis and was calculated for each group and for each time-point (i.e., Test 1, Test 2) separately (listed in Table 5). Global efficiency values of the cognitive-control networks (cingulo-opercular, fronto-parietal) for each group (children with RD, TRs) and for each testing time (Test 1, Test 2) were subjected to a 2 X 2 X 2 [Group (RD, TRs) X Network (cingulo-opercular, fronto-parietal) X Test (Test 1, Test 2)] RM-ANOVA and demonstrated a main effect of testing time [F(1,30) = 7.998, *P* = 0.008, η^2^ = 0.21], which suggests a greater global efficiency after intervention (Test 2) than before intervention (Test 1) regardless of group or network. No main effect for Group [F(1,30) = 0.016, *P* = 0.901, η^2^ = 0.001] or Network [F(1,30) = 2.054, *P* = 0.162, η^2^ = 0.64] was found. The RM-ANOVA did not find a Group X Test X Network interaction [F(1,30) = 0.393, *P* = 0.536, η^2^ = 0.013].

**Table 5:** Functional connectivity measures (global efficiency) for the cingulo-opercular and fronto-parietal networks before and after intervention in children with reading difficulty and typical readers.

Network	RD		TRs		*P* value	Contrast
	Test 1 (A)	Test 2 (B)	Test 1 (C)	Test 2 (D)		
Cingulo-opercular	0.86 (0.04)	0.91 (0.05)	0.87 (0.06)	0.90 (0.07)	-3.59*	B > A
					-1.462, ns	D>A
Fronto-parietal	0.86 (0.06)	0.88 (0.06)	0.85 (0.05)	0.88 (0.07)	-0.725 ns	B>A
					-1.084, ns	D>C

RD, children with RD (reading difficulty—dyslexia); TRs, typical readers; ns, not significant. Results are presented as mean (standard deviation).

*, significant (*P*<0.05);

ns, not significant.

Due to previous findings of the specific effect of training with the RAP on the activation and functional connectivity of the anterior cingulate cortex, which is part of the cingulo-opercular network, with other reading-related regions [35] [36], we aimed to determine the effect of the RAP training on resting-state functional connectivity in children with RD and TRs for each network separately. Global efficiency values were subjected to a 2 X 2 RM-ANOVA [Group (RD, TRs) X Test (Test 1, Test 2)] for the cingulo-opercular and fronto-parietal networks.

#### Cingulo-opercular network

A main effect of testing time [F(1,30) = 9.024, *P* = 0.005, η^2^ = 0.231] was found, suggesting an overall greater global efficiency of the cingulo-opercular network after intervention (Test 2) than before intervention (Test 1), regardless of reading group. No significant main effect for Group [F(1,30) = 0.033, *P* = 0.856, η^2^ = 0.001] or Group X Test [F(1,30) = 0.338, *P* = 0.565, η^2^ = 0.011] was found.

#### Fronto-parietal network

No significant main effect of Group [F(1,30) = 0.131, *P* = 072, η^2^ = 0.004], Test [F(1,30) = 1.633, *P* = 0.211, η^2^ = 0.52], or Group X Test [F(1,30) = 0.101, *P* = 0.753, η^2^ = 0.003] was found.

**Fig 1:**
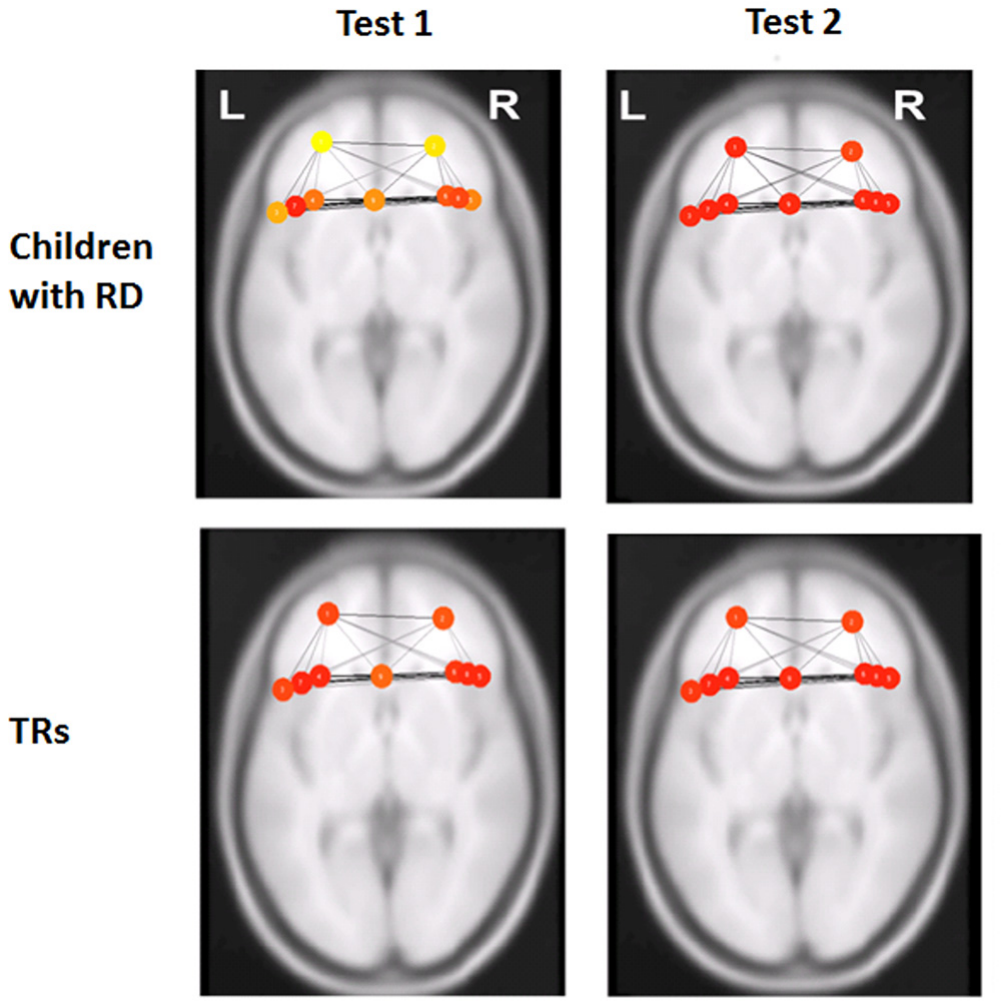
Greater functional connectivity in the cingulo-opercular network after training in children with RD. Functional connectivity measure (i.e., global efficiency) in the cingulo-opercular network before (Test 1) and after (Test 2) the reading intervention in children with reading difficulty (RD) and typical readers (TRs). Red color represents the highest global efficiency per node (beta = 0.9), the orange color represents the medium global efficiency per node (beta = 0.86) and the yellow color represents the lowest global efficiency per node (beta = 0.78) within the network. L, left and R, right.

**Fig 2:**
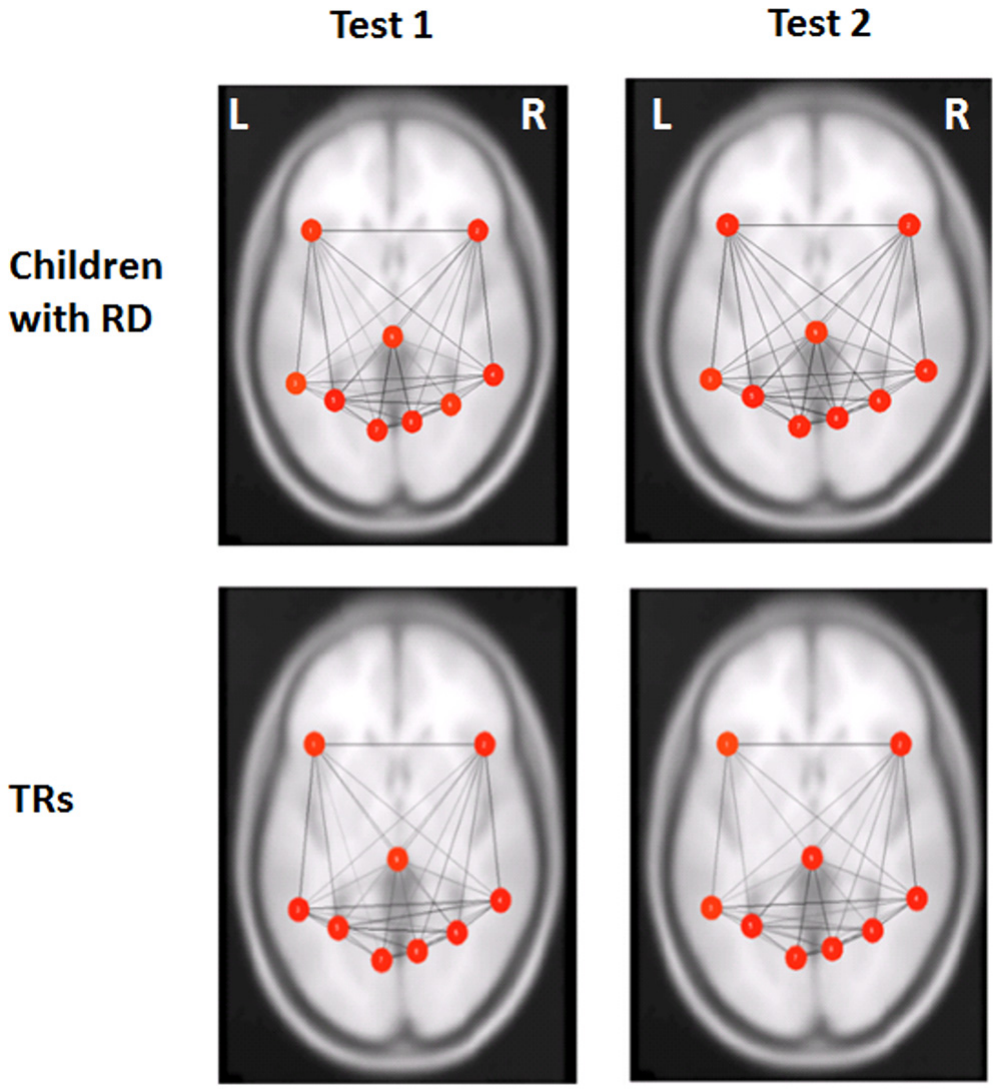
No change in global efficiency in the fronto-parietal network after training in children with RD and TRs. Functional connectivity measure (i.e., global efficiency) in the fronto-parietal network before (Test 1) and after (Test 2) the reading intervention in children with reading difficulty (RD) and typical readers (TRs). Red color represents the highest global efficiency per node, the orange color represents the medium global efficiency per node and the yellow color represents the lowest global efficiency per node within the network, represented by beta values. L, left and R, right.

**Fig 3:**
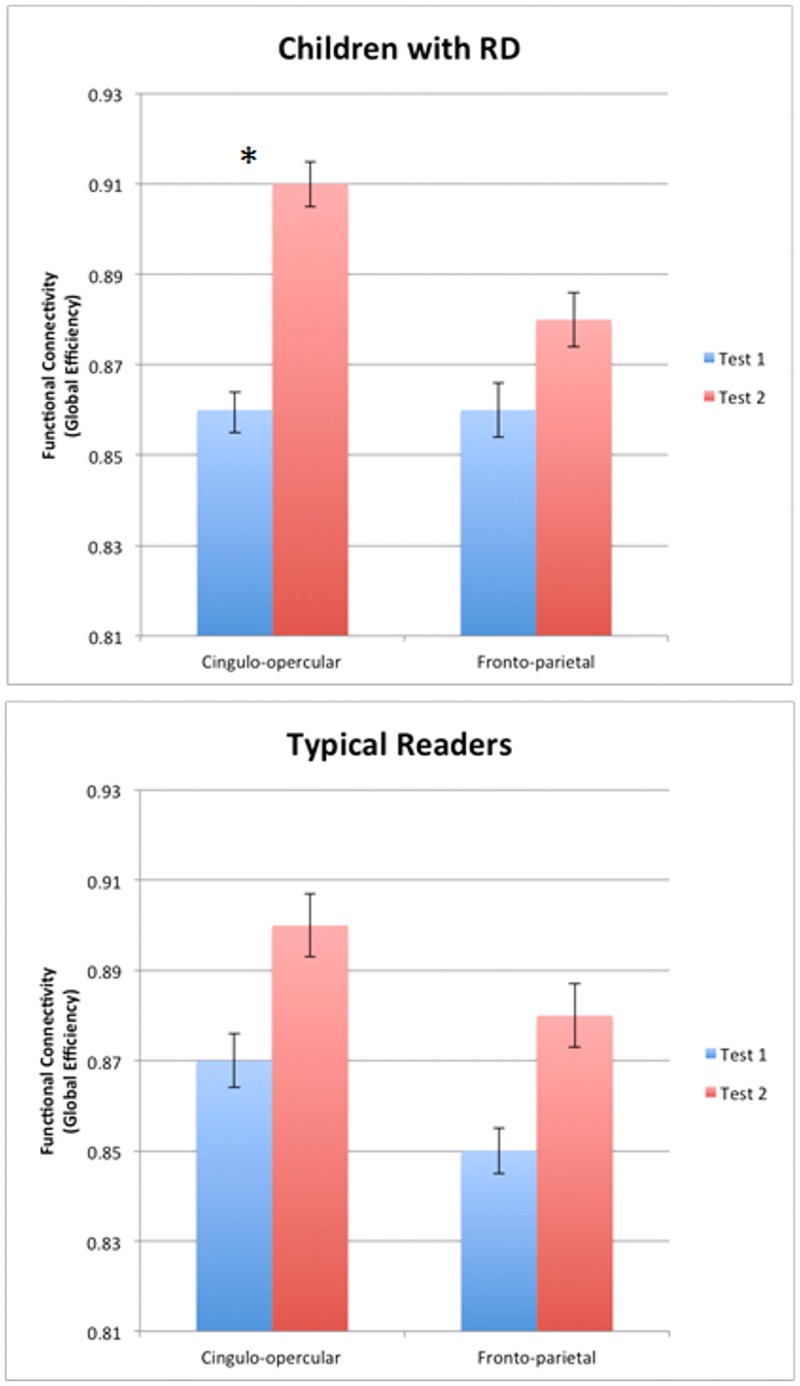
Significant change in global efficiency in the cingulo-opercular network in children with RD. Functional connectivity (i.e., global efficiency) in the cingulo-opercular (left bars) and fronto-parietal (right bars) networks in children with reading difficulty (RD, upper graph) and in typical readers (TRs, lower graph) before (Test 1; blue) and after (Test 2; red) the reading intervention. Error bars are included in the graphs. *P<0.05.

To assess whether one reading group or the other drove the increase in global efficiency following intervention (i.e., the overall Test 2 > Test 1 global efficiency), paired *t*-test analyses were performed within groups for each network separately. Children with RD had significantly increased global efficiency values in the cingulo-opercular network [t(14) = -3.59, *P* = 0.003], but not in the fronto-parietal network [t(14) = -0.725, *P* = 0.48]. Global efficiency in both networks in TRs did not exhibit significant differences (cingulo-opercular [t(16) = -1.462, *P* = 0.163]; fronto-parietal [t(14) = -1.085, *P* = 0.294]). See Fig 1 and Table 5 for the global efficiency values before and after intervention in the RD group and Fig 2 for those values for the TRs group (as well as S1 File). Fig 3 illustrates the changes in global efficiency in each network for both reading groups.

### Correlations between the gain in global efficiency and the gain in reading and EF behavioral measures

1. No significant correlations were found between the gain in global efficiency for the cingulo-opercular network and reading and EF measures.
2. No significant correlations were found between the gain in global efficiency for the fronto-parietal network and reading and EF measures.
3. The combined gain in global efficiency for the cingulo-opercular and fronto-parietal networks was correlated with greater reading scores only in children with RD (r = 0.521, *P*<0.05; scaled score of SWE and PWE from the TOWRE test).

**Fig 4:**
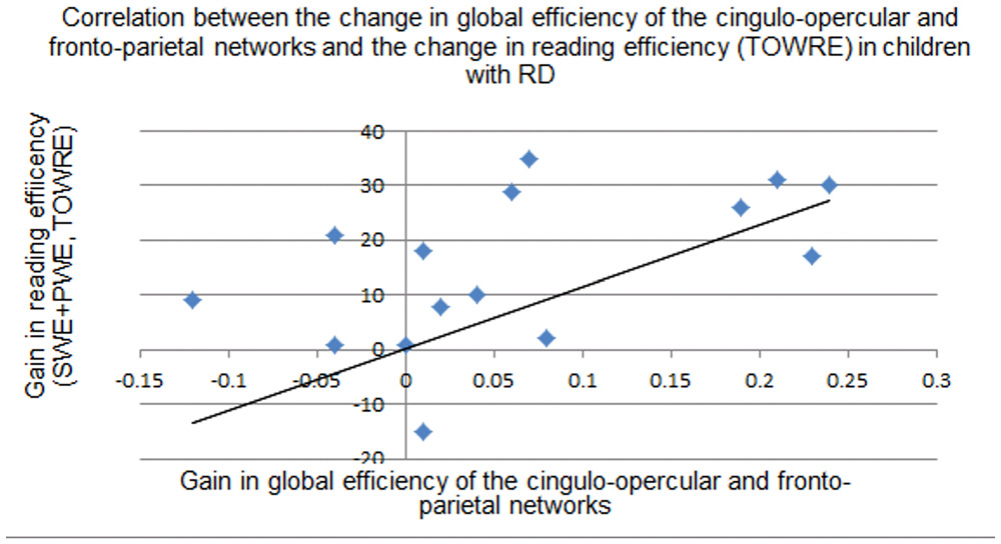
Scatter plot for the correlations between the change of global efficiency in both cingulo-opercular and fronto-parietal and reading efficiency in children with RD. The X axis represents the change in global efficiency in the cingulo-opercular and fronto-parietal in children with RD and the Y axis represents the gain in reading efficiency (SWE + PWE from the TOWRE) in scaled score.

The results suggest that greater gain in global efficiency for both networks was positively correlated with higher reading scores. See Fig 3 for the change in global efficiency for both networks in each reading group separately and Fig 4 for the correlations between the gain in both networks and reading measures.

## Discussion and Conclusions

The purpose of the current study was to examine the effect of the RAP training on functional connectivity (using global efficiency measures) of the fronto-parietal and cingulo-opercular cognitive-control networks in children with RD and TRs during rest. We also sought to determine the relationship between the change in functional connectivity and reading/EF behavioral measures. In addition to the positive effect of the RAP training on reading and EF measures, an overall increased global efficiency of the two cognitive-control networks was observed following training (i.e., main effect of Test, as measured before and after training). Training was found to have a specific effect on functional connectivity of the cingulo-opercular network. The combined change in global efficiency of both the cingulo-opercular and fronto-parietal networks was positively correlated with improved reading following the RAP training in children with RD.

### The positive effect of the RAP training on reading and executive functions

The participants in the current study showed greater reading abilities (word reading and overall reading efficiency, contextual fluency and comprehension) as well as improved EF [accuracy and speed of visual attention, speed of processing, shifting, reading accuracy (Wisconsin task) and speed (Stroop subtests), and inhibition] following the RAP training. These results, which have been repeatedly observed in other orthographies (e.g., Hebrew; see [33]), confirm our suggestion that the RAP affects more basic abilities than reading per-se. We have previously suggested that the RAP manipulation forces the reader to focus visual attention and process the graphemes in a speeded manner. Since the training program encourages the reader to process more given letters within a time-constraint, speed of processing is challenged and visual attention is focused to track the deleted letters and therefore, fluency improves. The need to switch between the natural attempts of children with RD to decode the words to more holistic reading may be triggered by this manipulation, since it does not allow the participants enough time to decode each word and “pushes” them to move forward with their reading. Although this may be the reason for the improved switching ability, an eye-movement study that tracks the number and length of fixations per word after training would be needed to confirm this point.

We have claimed that the effect of the RAP training is mediated mainly through higher-order cognitive-control abilities (i.e., EF), which has been confirmed by other neuroimaging studies indicating a greater functional connectivity between error-detection regions (i.e., anterior cingulate cortex) and regions related to reading (i.e., fusiform gyrus) (see [18] for a model of reading networks and [68]). These previous studies were based on task-related data and were also observations during rest [44]. However, this association does not address causality, and further study using Dynamic Causal Modeling for effective connectivity to examine this point in depth is warranted.

### Are the reading and executive function improvements mediated by an effect on the cognitive-control networks?

Results of the current study indicate an overall effect of the RAP training on functional connectivity (i.e., global efficiency measures) of the cognitive-control networks (i.e., main effect of Test). This finding supports our original hypothesis that the RAP training has an overall effect on more basic abilities, even in the absence of a task (i.e., during rest). However, despite the overall effect of testing time on functional connectivity of the two cognitive-control networks, only children with RD showed a significantly increased functional connectivity within the cingulo-opercular network (calculated using global efficiency measures). As noted, the anterior cingulate cortex, which is part of the cingulo-opercular network [22],was previously found to have increased activation [36] and increased Error Related Negativity amplitudes during reading tasks after the RAP training in both children [33] and adults [42]. Increased functional connectivity between the visual cortex (i.e., fusiform gyrus) and the anterior cingulate cortex also has been observed after the RAP training during rest [44]. We therefore suggest that these findings may indicate a greater synergy between the VWFA (i.e., fusiform gyrus [69]) and error detection and EF regions. One possibility is that the RAP training affects the VWFA by stimulating it to result in reading words more “holistically” and therefore improving the mental lexicon that in turns results in increased error-detection activation in the case of an erroneous reading. Another option is that the RAP has a direct effect on EF and error monitoring since this program has EF elements implemented within it (visual attention, speed of processing, and working memory), and this is a remaining question. By training both the EF and the error-monitoring system, the child is capable of detecting reading errors more efficiently and therefore, reading improves and error monitoring during reading errors improves. Since the current study shows greater functional connectivity in the cingulu-opercular network, even during rest, this may suggest that the effect of the RAP is directly on the EF, and that this direct effect may drive the improvement in reading.

Our attempt to connect the change in functional connectivity to reading improvement revealed a significant correlation between *the combined gain* in functional connectivity of the cingulo-opercular *and* the fronto-parietal networks and an overall reading improvement in children with RD. Despite the critical role of the cingulo-opercular network in reading and reading improvement, children with RD may need a combined increase in connectivity to overcome their difficulties with reading. Another possibility is that due to the speed-of-processing manipulation of the RAP training, not only does functional connectivity increase, but also the synchronization between the activation of the cognitive-control networks improves following training, which is based on the assumed role of the fronto-parietal network in speed of processing [22]. Due to the temporal limitations of MRI, a simultaneous fMRI-EEG acquisition (with its excellent temporal resolution) would be useful to address this point.

In the current study, TRs did not show significant correlation between the change in functional connectivity and reading improvement. We have found that the RAP seems to be more useful for readers whose baseline measures are lower [33, 35, 36], which may explain why the change in functional connectivity in TRs was not significant. An alternative explanation is that the increased error monitoring observed in TRs [33]] is secondary for a primary effect on a different network. Although two central cognitive-control networks have been proposed by Dosenbach and colleagues to be involved, the cingulo-opercular and fronto-parietal networks [22], other networks that are part of cognitive control may be primarily affected by the RAP training in TRs [70]. A future study examining the effect of the RAP training on functional connectivity of the dorsal attention and default-mode networks in TRs and individuals with RD should be performed, as well as testing the effect of longer or more intensive intervention in these groups on the two cognitive control networks.

To conclude, the current study provides evidence for the effect of the RAP training, an EF-based reading intervention, on functional connectivity of cognitive-control networks during rest in children with reading difficulties. These results are encouraging because they highlight the importance of these cognitive-control networks in individuals with RD, which supports other reports of involvement of cognitive-control networks in other disorders (e.g., [70]]). It may be interesting to examine the functional connectivity of the cingulo-opecular and fronto-parietal networks and reading impairments in other populations with RD, since this might serve as a biomarker for reading impairments. Another implication of our results may be a support of including an EF-based intervention at a very young age, even before reading is acquired, for children who are at-risk to develop RD (e.g., due to a familial risk for dyslexia or other RD causes). This approach might minimize the accumulated RD after reading is acquired and help facilitate reading acquisition. The ability to relate reading improvement to a change in cognitive-control networks during rest may enable the detection of the effectiveness of an academic intervention without an active collaboration with the participant. Reading acquisition might also be facilitated even before it is officially acquired (e.g., in preschool), by also training EF in typically developing children. Another interesting possibility for a future implication, which we could not achieve due to a low sample size, is to estimate the profile of children who may benefit more from the RAP training. With a larger number of participants, a support vector machine of baseline EF and reading measures together with the change in global efficiency of the fronto-parietal and cingulo-opercular, will enable development of a model of the relationship between a change in cognitive-control networks and behavioral measures (after [71]). Pending a large sample of participants, we will be able to predict who may gain more from the RAP training based on baseline behavioral measures.

Our conclusions have been made with consideration of the following study limitations: The model we based our analysis on is based on networks derived from a parcellation method developed by Gordon and colleagues [72]. This method defines the ‘parcels’ based on boundaries defined by the data itself and includes a very rigorous motion-correction approach [72, 73]. Therefore, a future study employing the method described in these papers may deepen our understanding of additional networks that may be involved in reading remediation (i.e., visual attention, default mode network, see [18, 19] [23]) in typically developing children as well as those with reading difficulties. It is also important to note that the positive correlation found between the change in functional connectivity and reading measures was only evident when using the TOWRE reading task. This task is a word-level reading fluency task, which does not asses contextual reading and neither fluency nor comprehension. This may be attributed to the relatively low number of datasets or a longer resting-state condition (with a larger number of frames per child) or due to a need for a longer intervention in order to find an effect on these measures. A future study that examines the effect of these variables on the functional connectivity of the cognitive control networks is needed.

## Supporting Information

S1 File The change in global efficiency for the cingulo-opercular and fronto-parietal networks after intervention. (XLSX)
